# Supplementary material for: Mechanisms governing the pioneering and redistribution capabilities of the non-classical pioneer PU.1
Source: Nat Commun. 2020 Jan 21;11:402. doi: 10.1038/s41467-019-13960-2 (PMC6972792; doi:10.1038/s41467-019-13960-2)
Supplement: Supplementary file 7 — Source data [file 41467_2019_13960_MOESM7_ESM.zip › Source_Data/Figure6/Figure6B_MotifScanOutput/ETS-specific/homerResults/motif47.similar.html]

motif47

## Information for motif47

A
C
G
T
T
G
A
C
A
G
C
T
G
A
T
C
T
A
C
G
A
T
G
C
T
A
C
G
T
G
C
A
T
C
A
G
T
G
C
A
A
C
T
G
A
G
C
T
  
Reverse Opposite:  

T
C
G
A
T
G
A
C
A
C
G
T
A
G
T
C
A
C
G
T
A
T
G
C
T
A
C
G
A
T
G
C
C
T
A
G
T
C
G
A
A
C
T
G
T
C
G
A
  

|  |  |
| --- | --- |
| p-value: | 1e-16 |
| log p-value: | -3.861e+01 |
| Information Content per bp: | 1.700 |
| Number of Target Sequences with motif | 69.0 |
| Percentage of Target Sequences with motif | 0.90% |
| Number of Background Sequences with motif | 84.1 |
| Percentage of Background Sequences with motif | 0.21% |
| Average Position of motif in Targets | 98.1 +/- 56.8bp |
| Average Position of motif in Background | 104.2 +/- 61.0bp |
| Strand Bias (log2 ratio + to - strand density) | -0.2 |
| Multiplicity (# of sites on avg that occur together) | 1.06 |
| Motif File: | file (matrix) reverse opposite |

### Similar de novo motifs found

|  |  |  |  |  |  |  |  |
| --- | --- | --- | --- | --- | --- | --- | --- |
| Rank | Match Score | Redundant Motif | P-value | log P-value | % of Targets | % of Background | Motif file |
| 1 | 0.877 | A C G T G T A C C A G T A G T C A C T G A G T C A C T G C G T A | 1e-15 | -34.652671 | 1.25% | 0.42% | motif file (matrix) |
| 2 | 0.976 | A G C T A G T C A C G T G T A C A T C G G T A C C A T G T G C A A T C G T G C A A T C G | 1e-15 | -34.578025 | 0.98% | 0.27% | motif file (matrix) |
| 3 | 0.818 | G A C T T C A G C A G T T A G C A C T G G T A C C A T G T G A C A T C G G T C A | 1e-13 | -30.220123 | 1.05% | 0.34% | motif file (matrix) |
| 4 | 0.949 | C T A G G T A C A C G T T A G C A G C T A G T C C A T G A T G C A C T G T C G A A T C G C T G A A C T G | 1e-12 | -28.822359 | 0.68% | 0.16% | motif file (matrix) |
